# Supplementary material for: (2-Hydroxy-3-Methoxybenzylidene)thiazolo[3,2-a]pyrimidines: Synthesis, Self-Assembly in the Crystalline Phase and Cytotoxic Activity
Source: Int J Mol Sci. 2023 Jan 20;24(3):2084. doi: 10.3390/ijms24032084 (PMC9917025; doi:10.3390/ijms24032084)
Supplement: Supplementary file 1 [file ijms-24-02084-s001.zip › ijms-2161001-supplementary.pdf]

Supplementary Materials

(2-Hydroxy-3-Methoxybenzylidene)thiazolo[3,2-*a*]pyrimidines: Synthesis, Self-Assembly in the Crystalline Phase and Cytotoxic Activity

Artem S. Agarkov [1], Anna A. Nefedova [1], Elina R. Gabitova [2], Dilyara O. Mingazhetdinova [2], Alexander S. Ovsyannikov [1], Daut R. Islamov [3], Syumbelya K. Amerhanova [1], Anna P. Lyubina [1], Alexandra D. Voloshina [1], Igor A. Litvinov [1], Svetlana E. Solovieva [1,*] and Igor S. Antipin [1]

1 Arbuzov Institute of Organic and Physical Chemistry, FRC Kazan Scientific Center, Russian Academy of Sciences, Arbuzova 8, 420088 Kazan, Russia
2 Kazan Federal University, A.M. Butlerov Chemical Institute, 18 Kremlevskaya St., 420008 Kazan, Russia
3 Laboratory for Structural Studies of Biomacromolecules, FRC Kazan Scientific Center of RAS, 2/31 Lobachevskogo Str., 420111 Kazan, Russia
* Correspondence: evgersol@yandex.ru

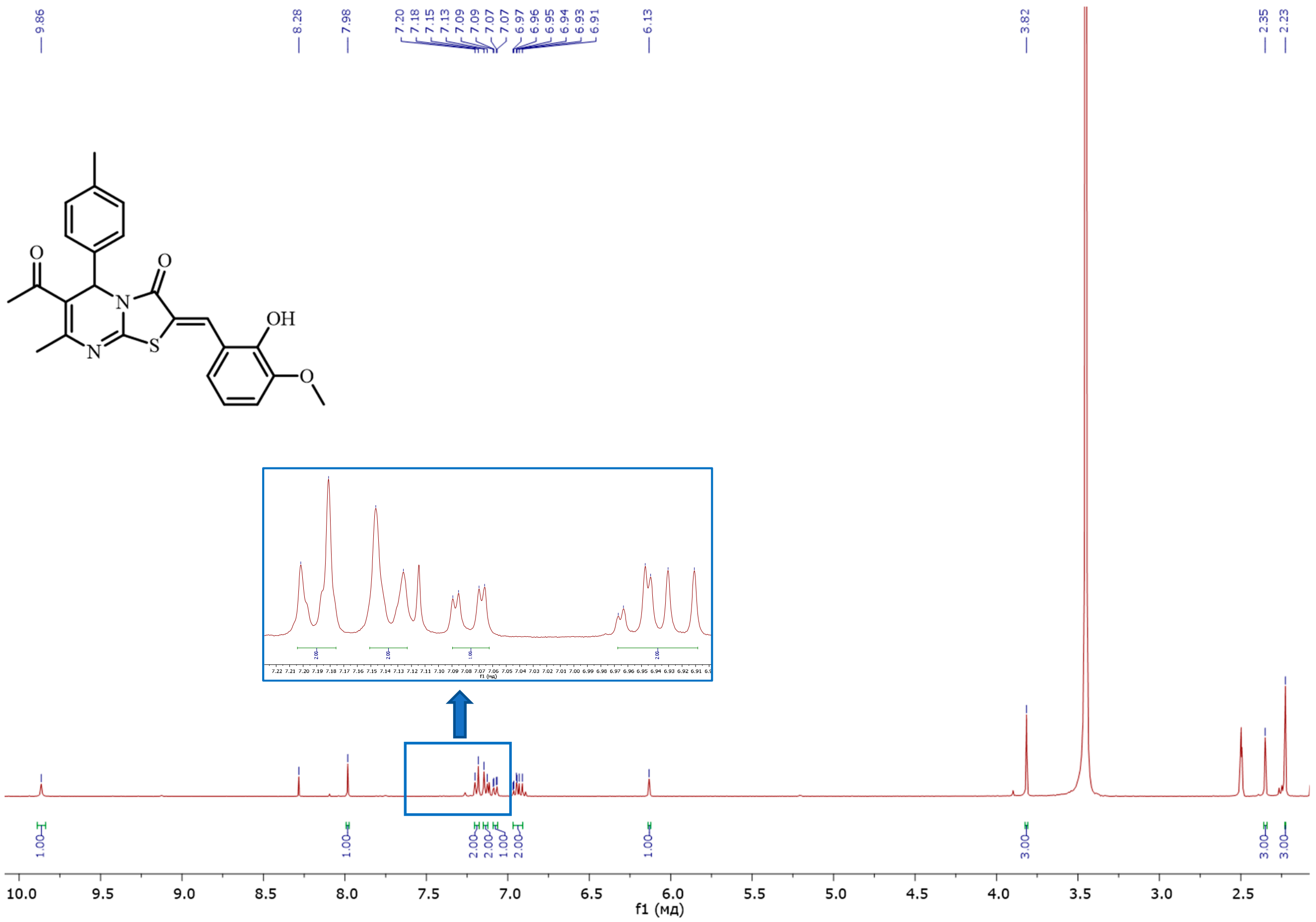

Figure S1. ^{1}H NMR spectrum of compound **1** (DMSO-d$_6$, 500 MHz, 25^{0}C).

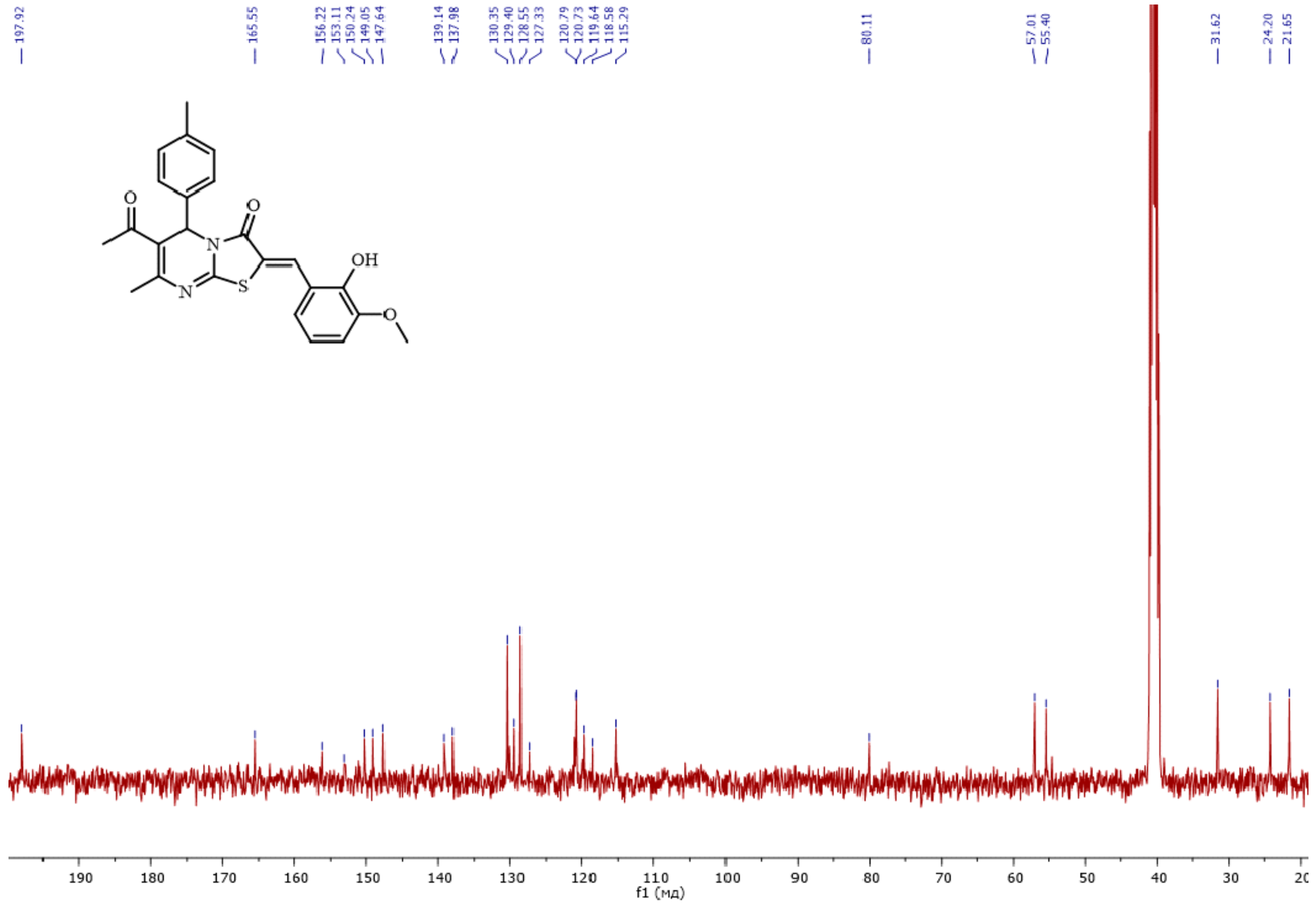

Figure S2. ^{13}C NMR spectrum of compound **1** (DMSO-d_6, 100 MHz, 25^{0}C).

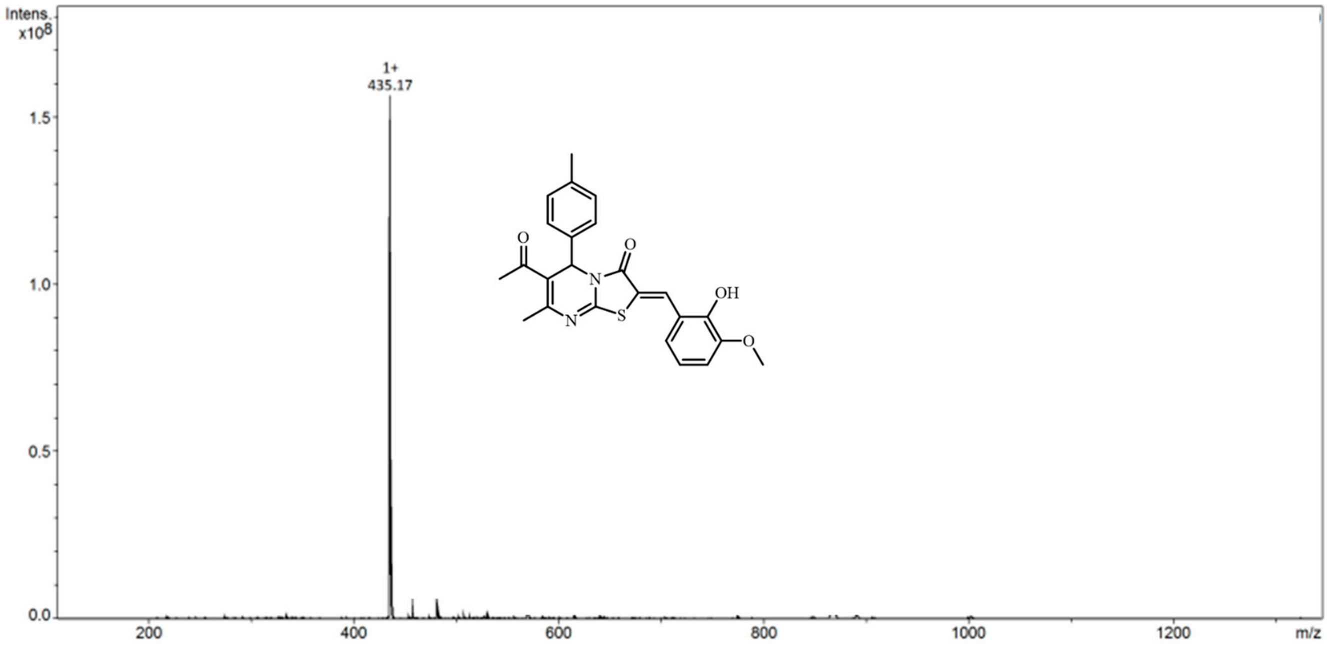

Figure S3. ESI MS spectrum of compound **1** (Ion Polarity: Positive).

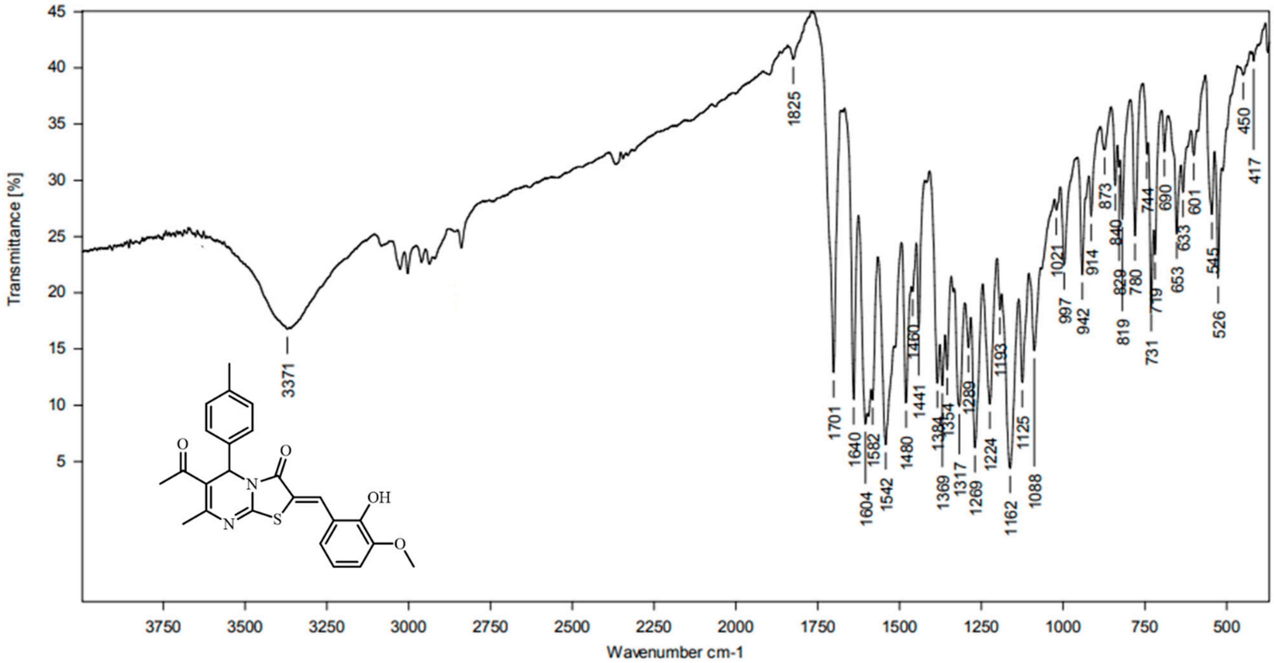

Figure S4. IR spectrum of compound **1** (KBr tablet).

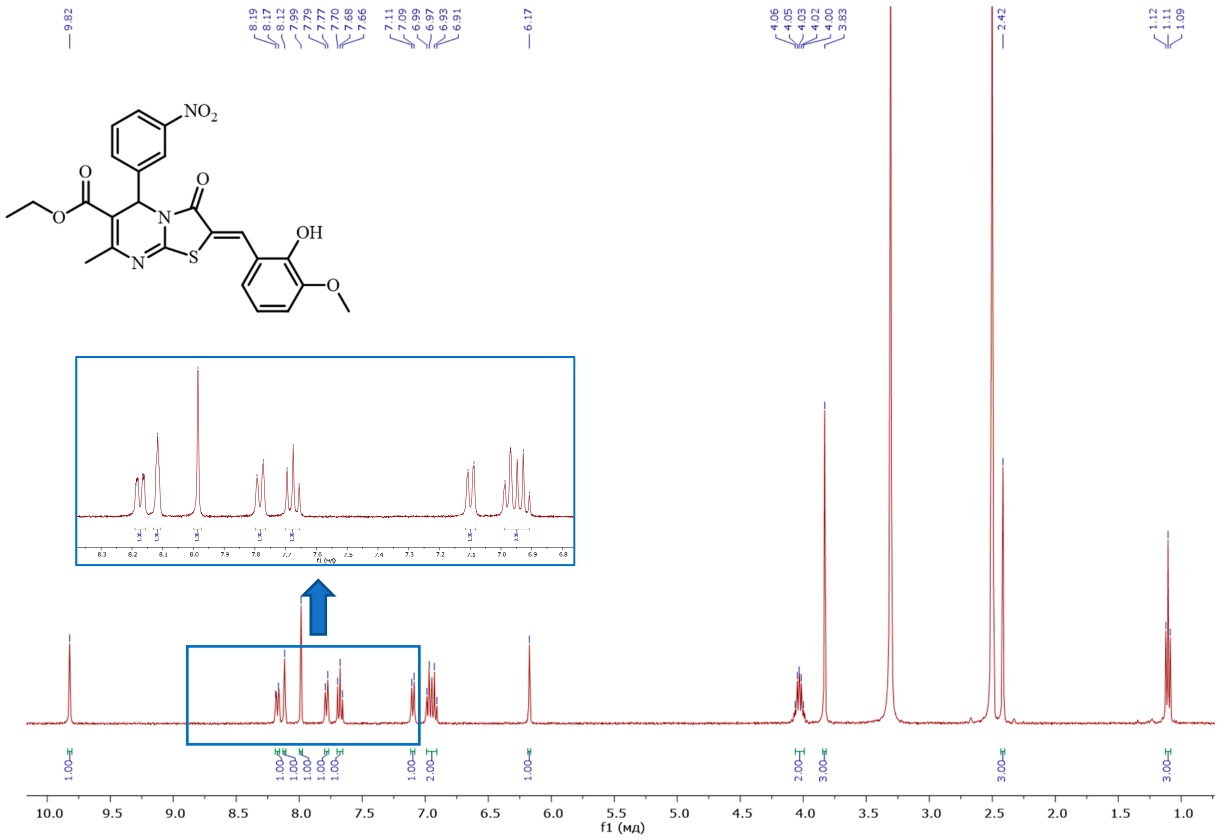

Figure S5. ^{1}H NMR spectrum of compound **2** (DMSO-d$_6$, 500 MHz, 25^{0}C).

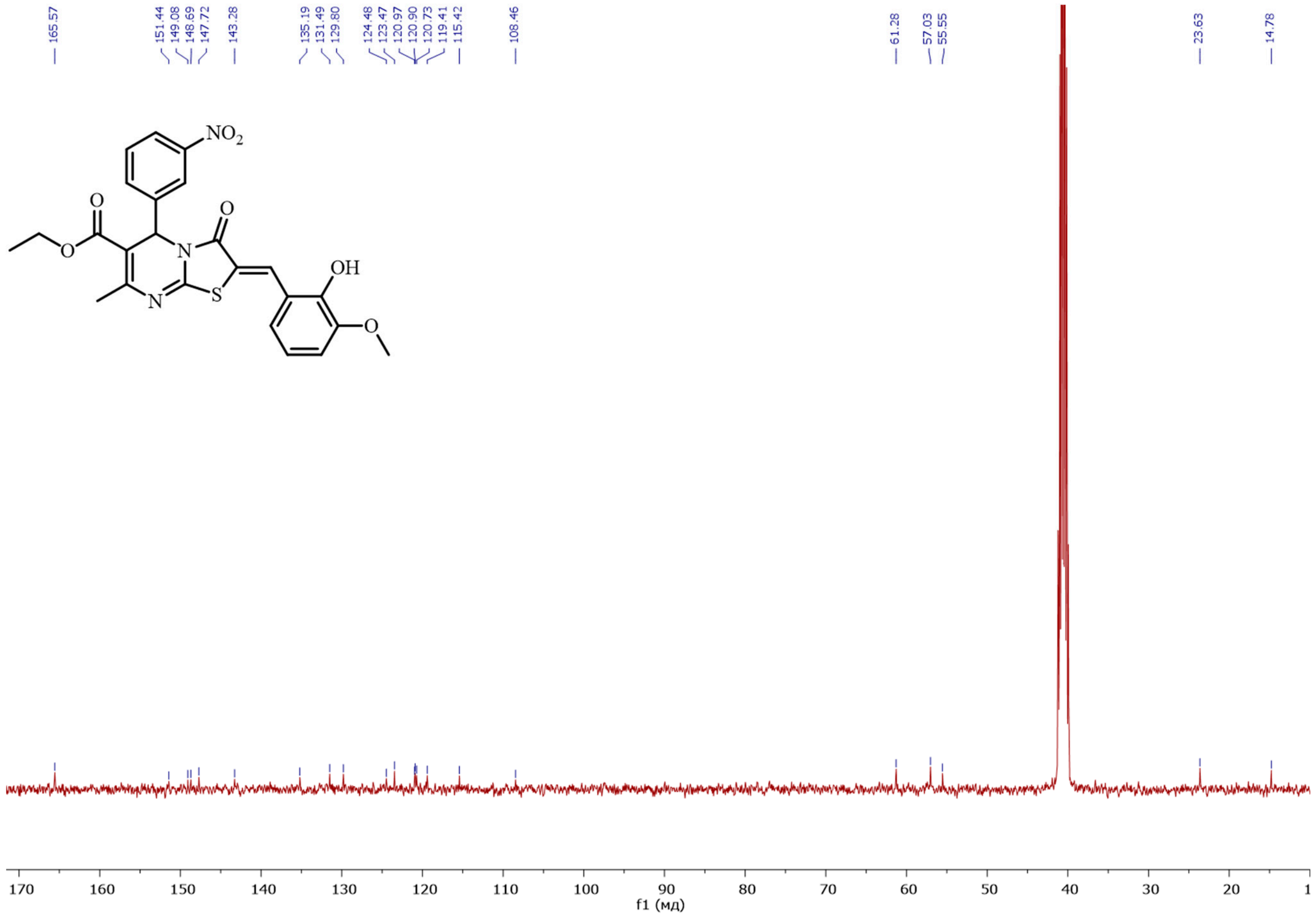

Figure S6. ^{13}C NMR spectrum of compound **2** (DMSO-d_6, 100 MHz, 25^{0}C).

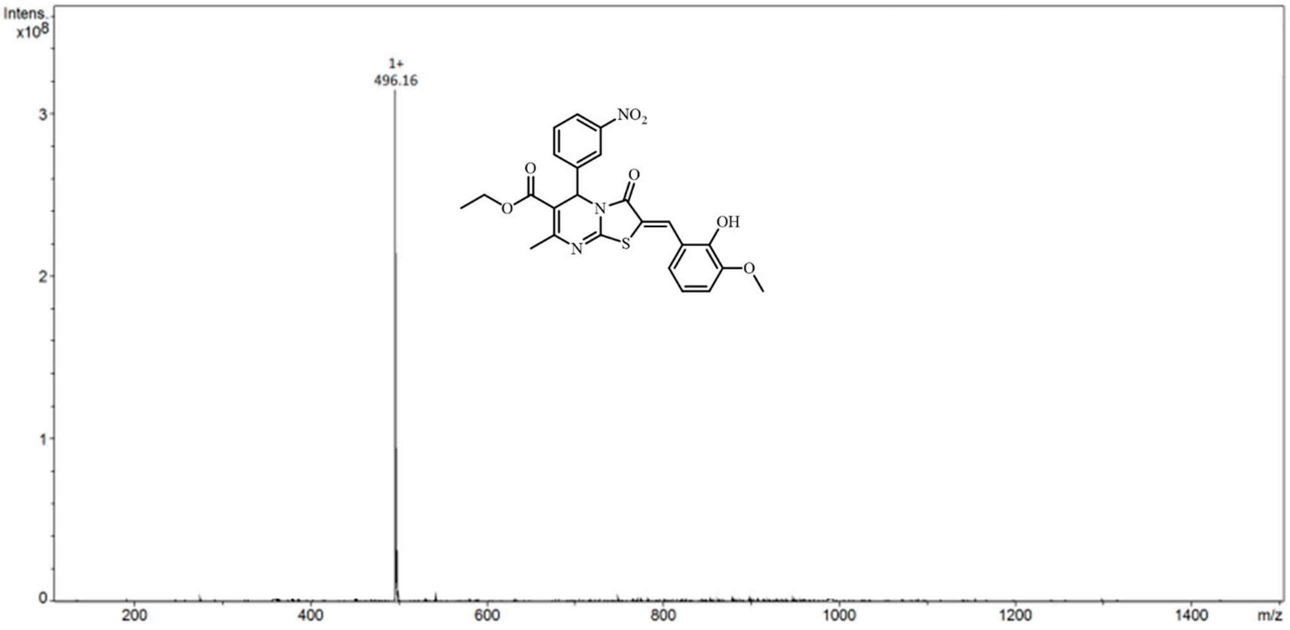

Figure S7. ESI MS spectrum of compound **2** (Ion Polarity: Positive).

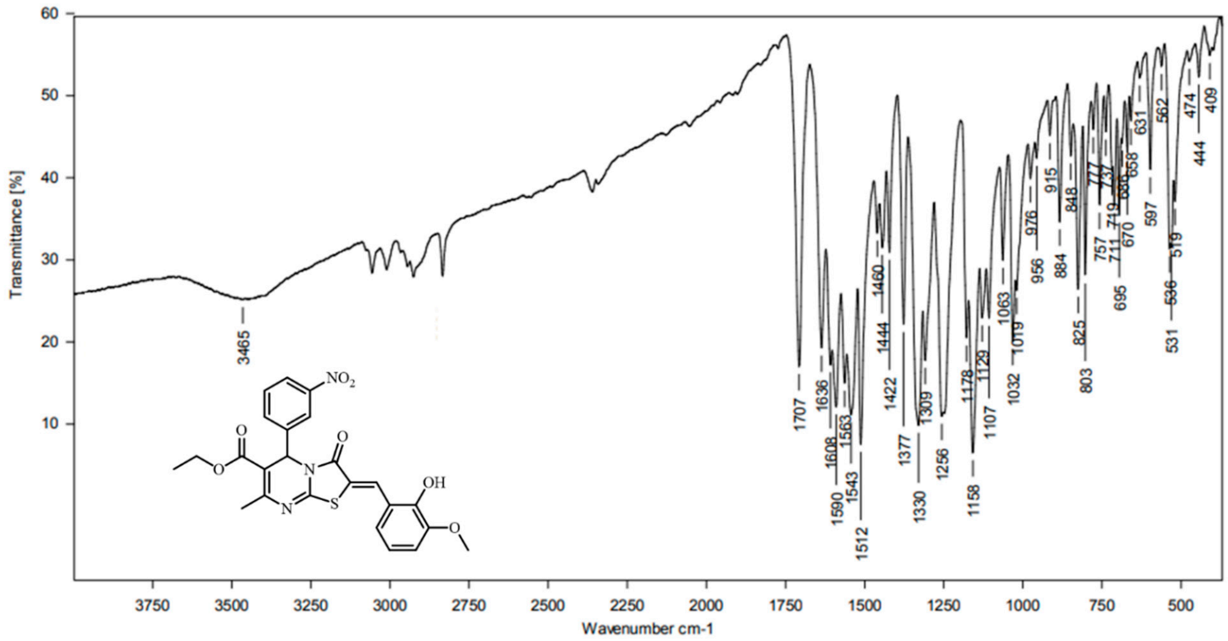

Figure S8. IR spectrum of compound **2** (KBr tablet).

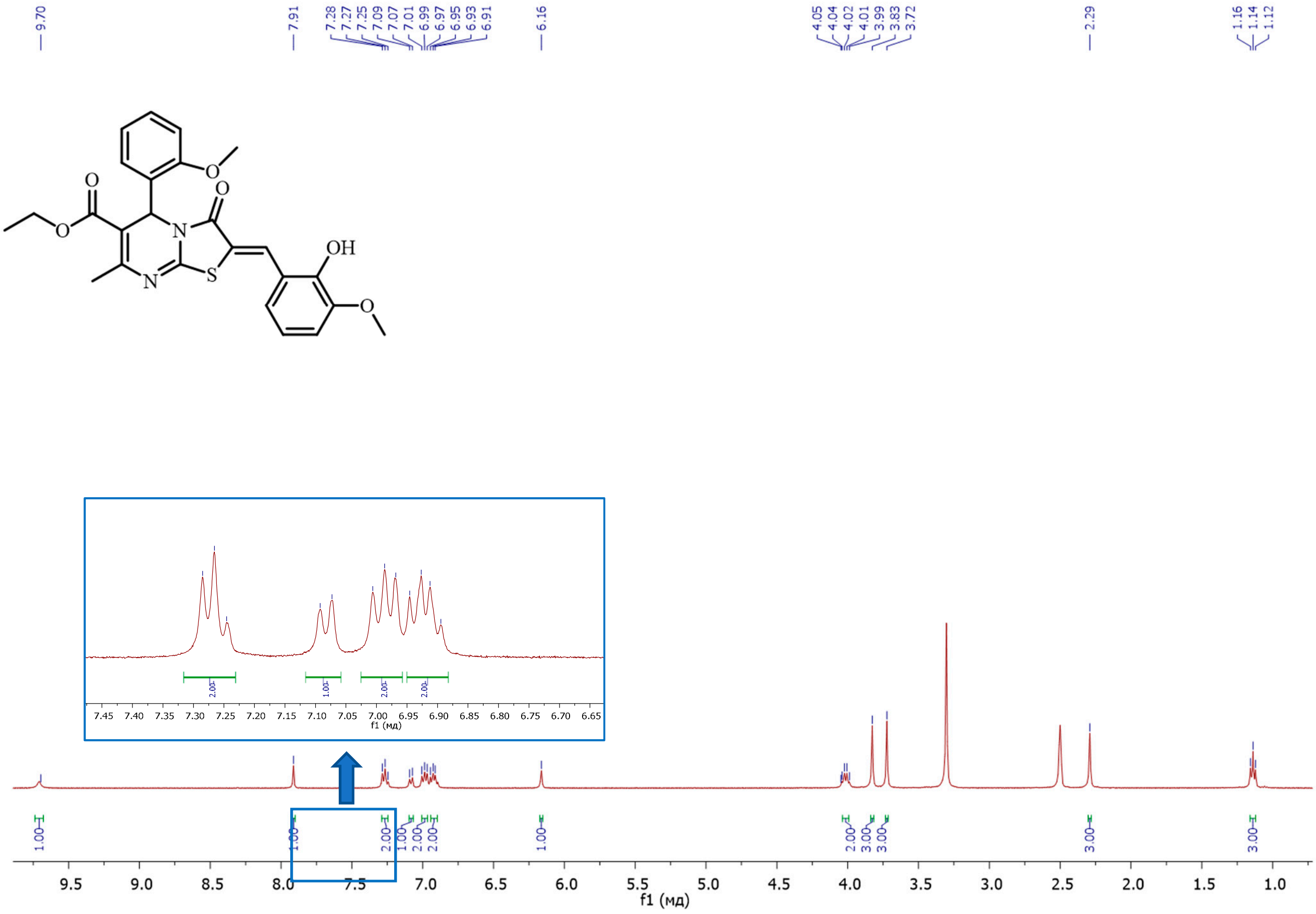

Figure S9. ^{1}H NMR spectrum of compound **3** (DMSO-d6, 500 MHz, 25⁰C).

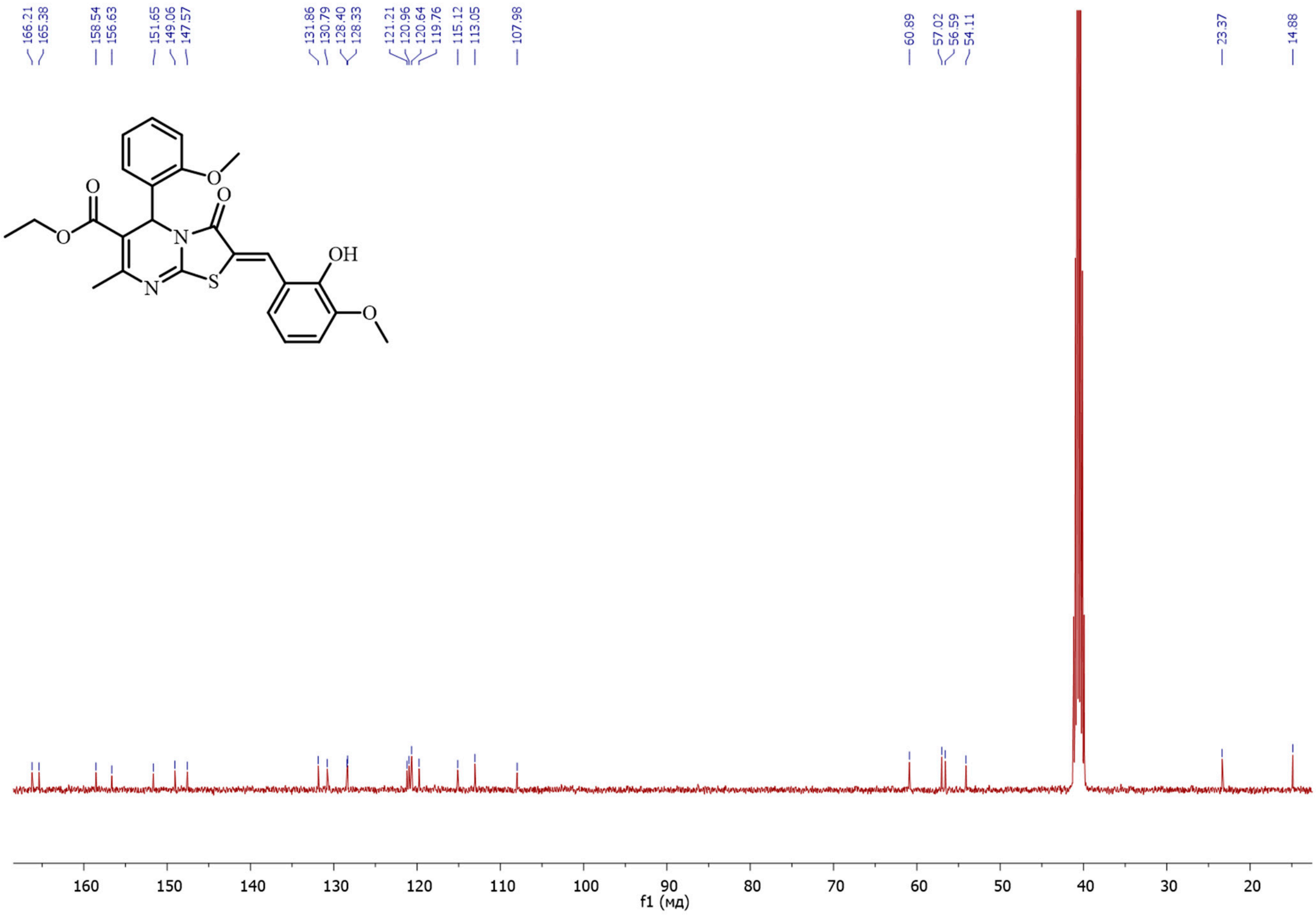

Figure S10. ^{13}C NMR spectrum of compound **3** (DMSO-d_6, 100 MHz, 25^{0}C).

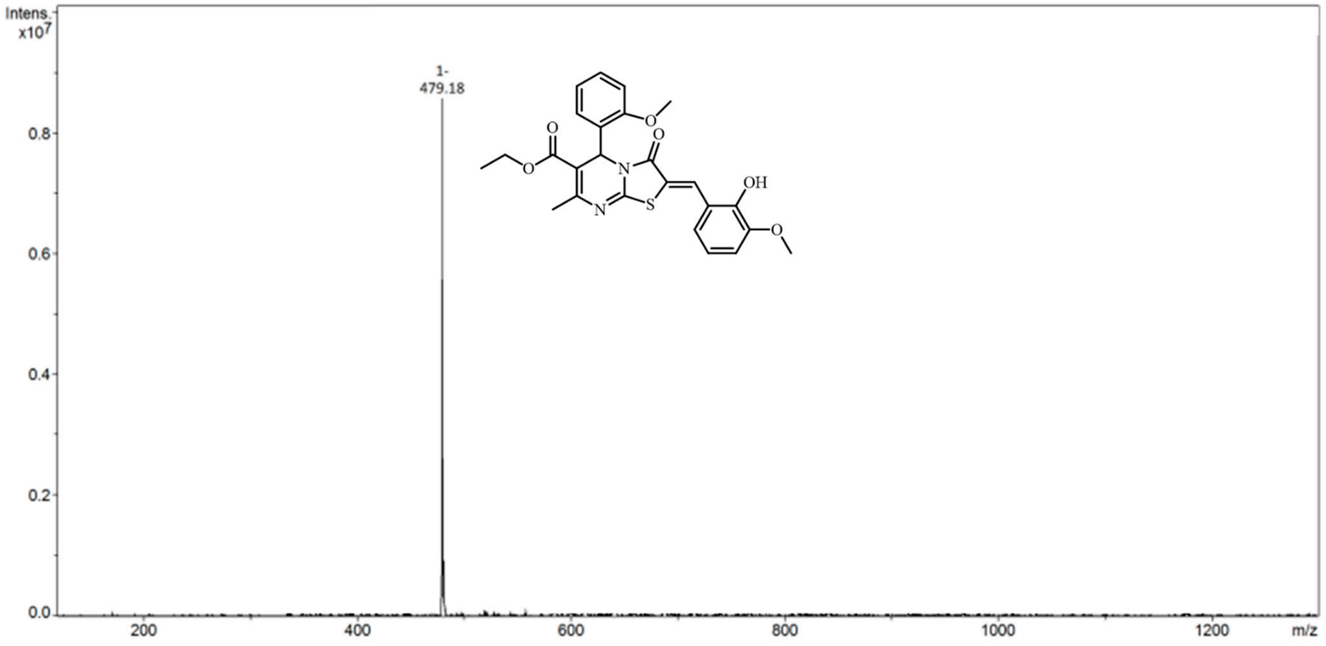

Figure S11. ESI MS spectrum of compound **3** (Ion Polarity: Negative).

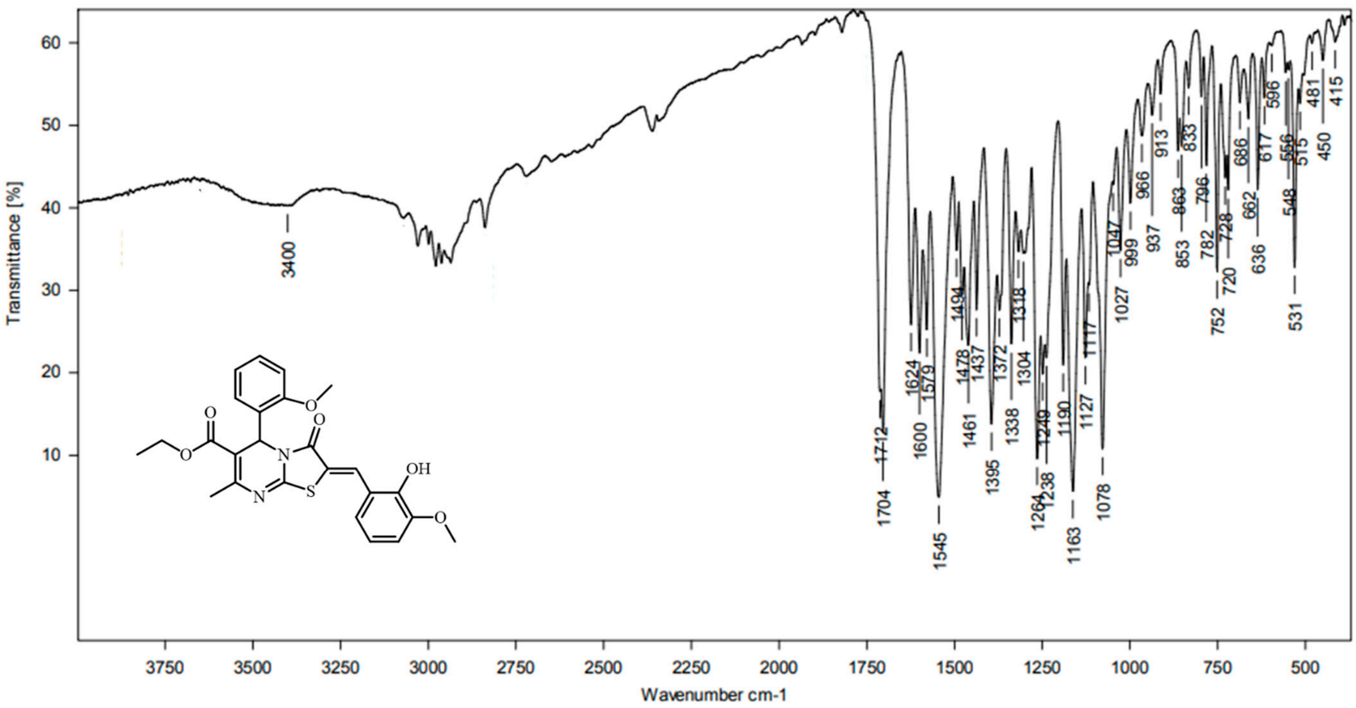

Figure S12. IR spectrum of compound **3** (KBr tablet).

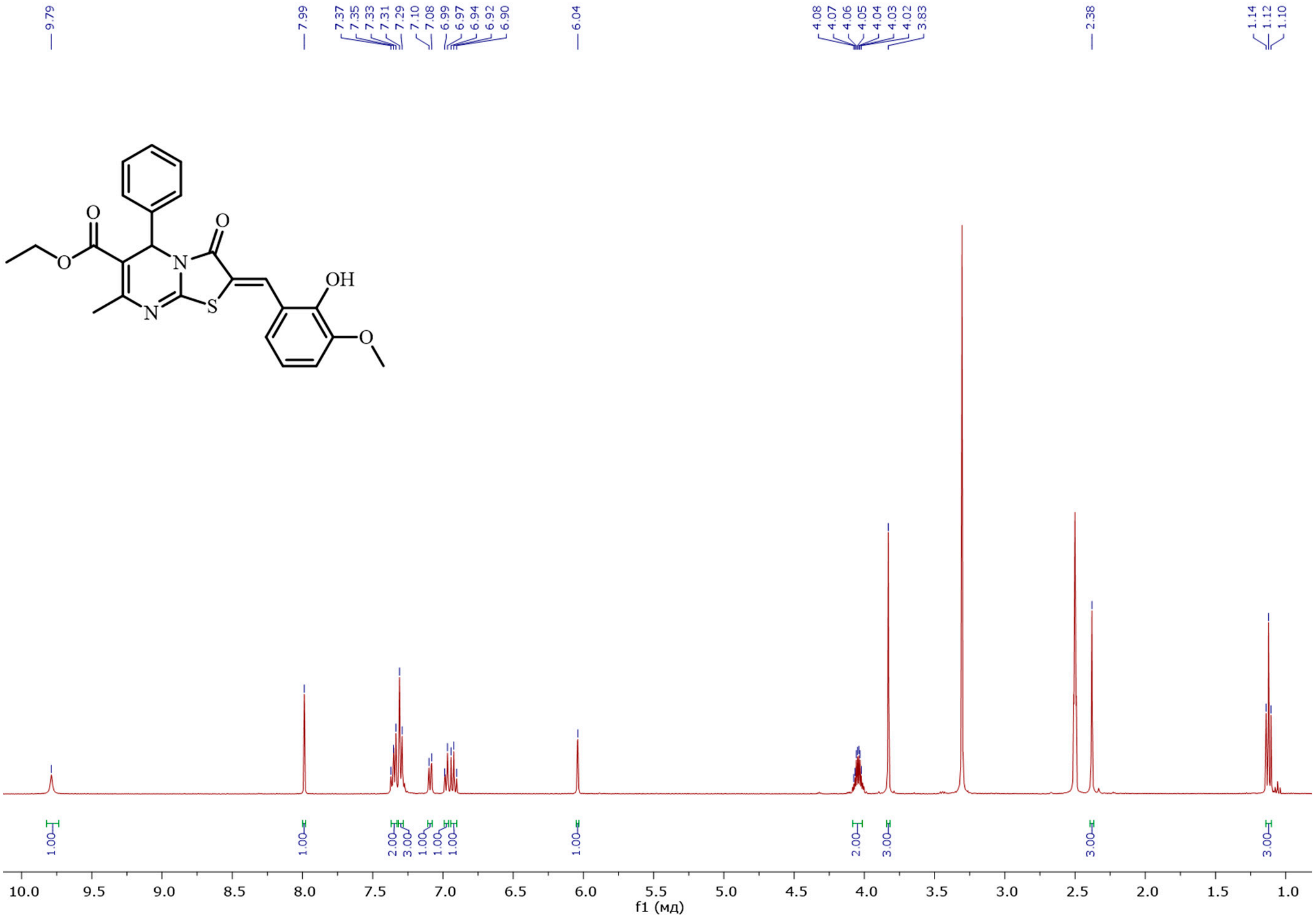

Figure S13. ^{1}H NMR spectrum of compound **4** (DMSO-d6, 500 MHz, 25⁰C).

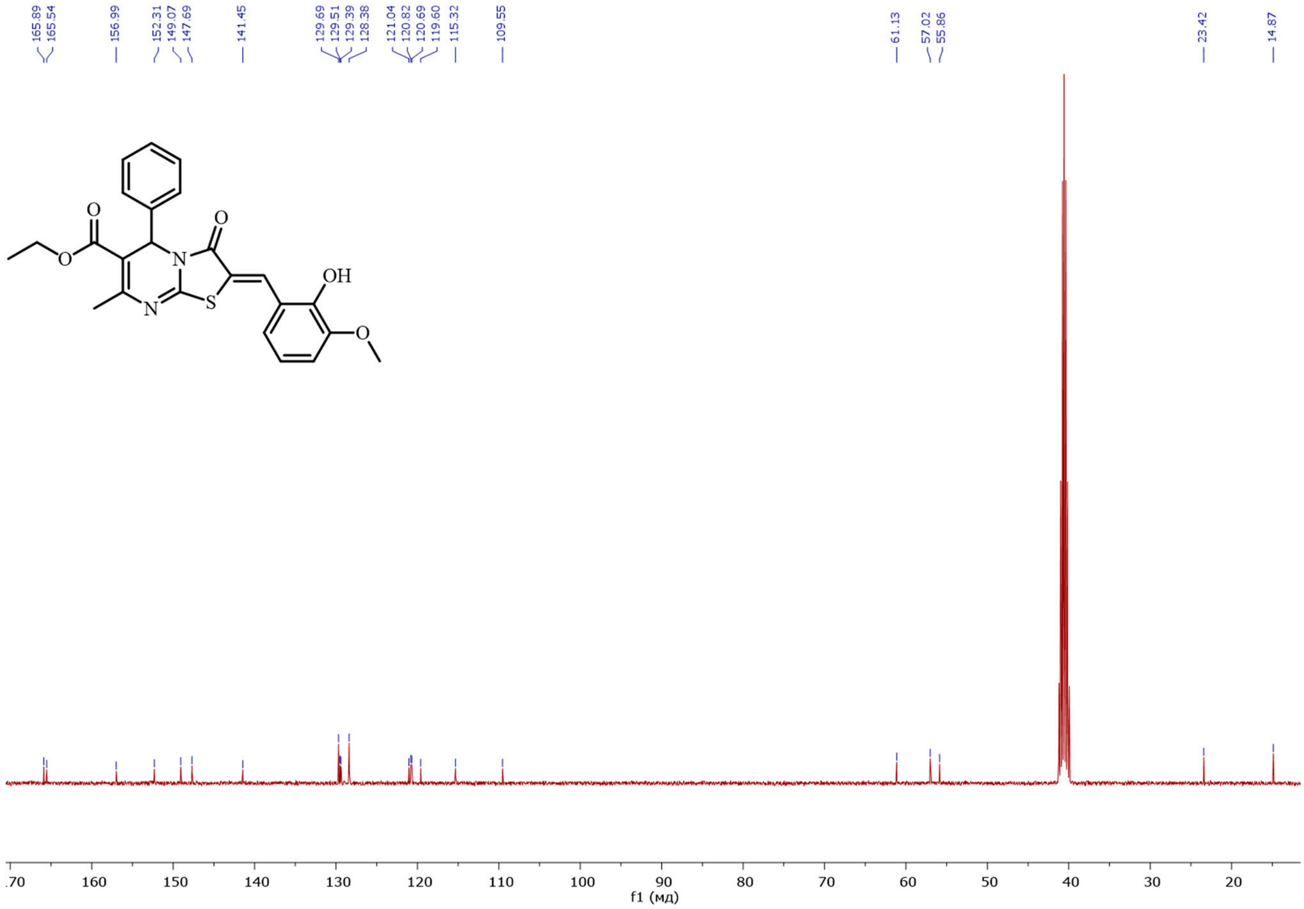

Figure S14. ^{13}C NMR spectrum of compound **4** ($DMSO-d_6$, 100 MHz, 25^{0}C).

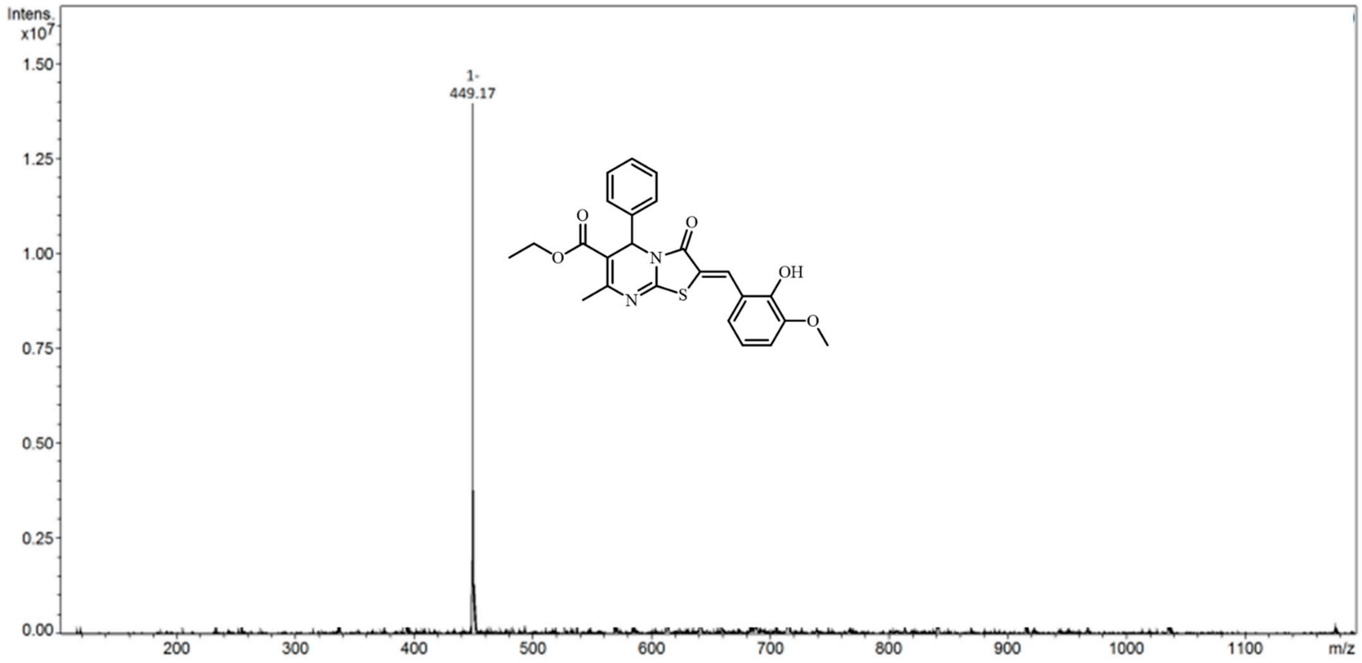

Figure S15. ESI MS spectrum of compound **4** (Ion Polarity: Negative).

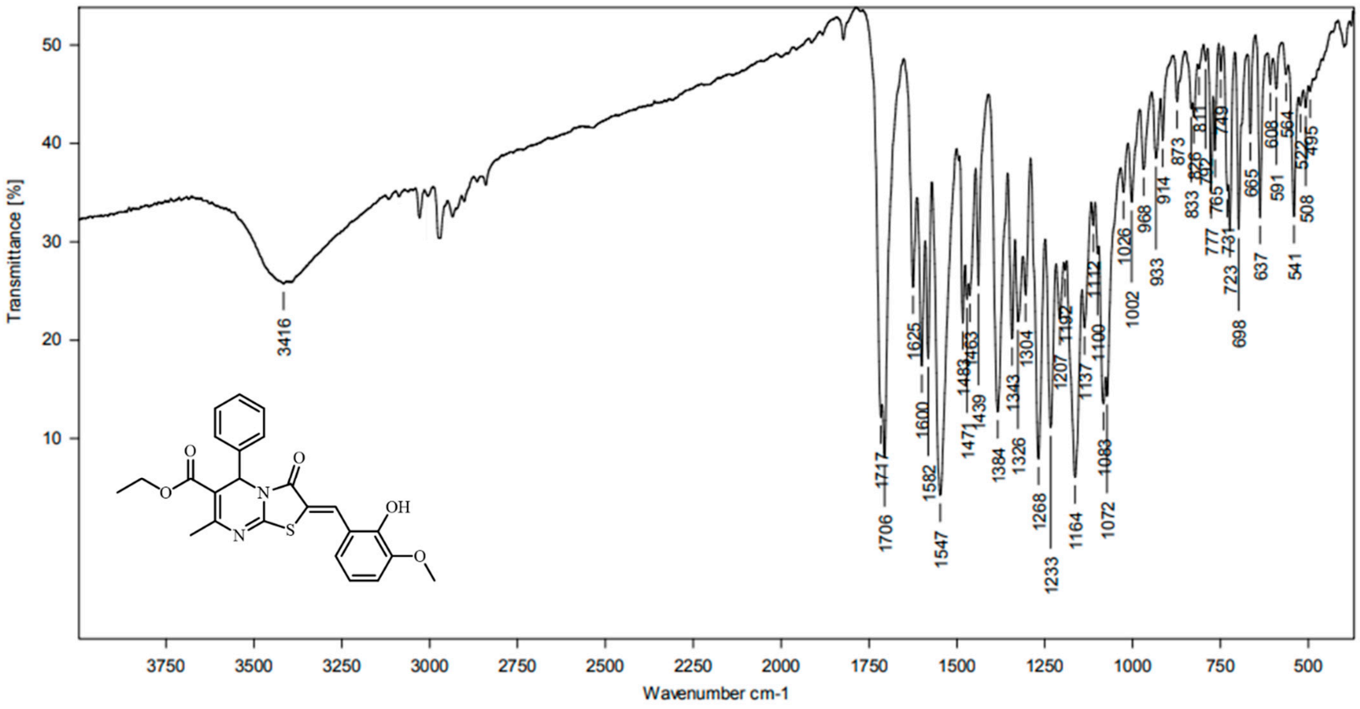

Figure S16. IR spectrum of compound **4** (KBr tablet).

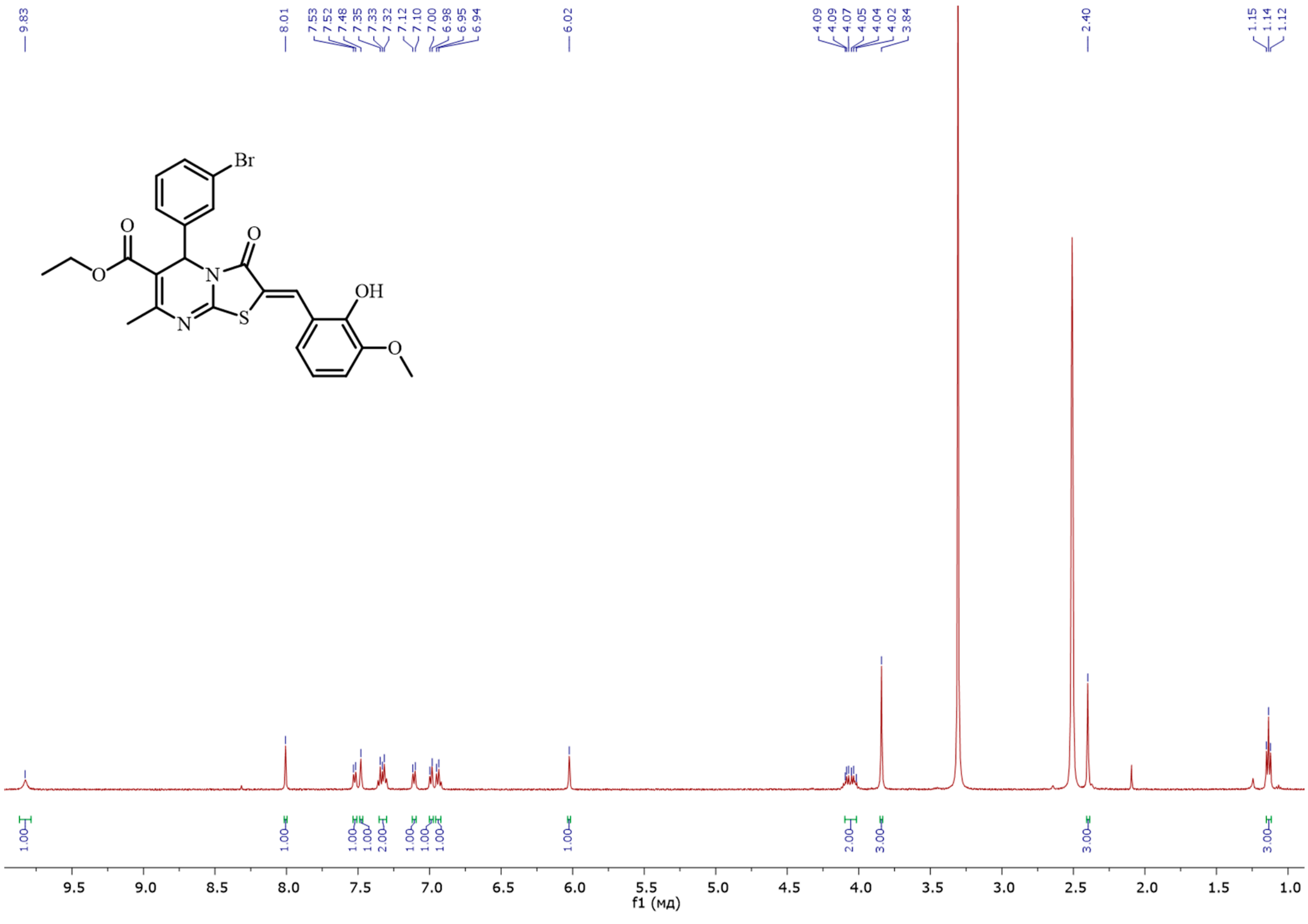

Figure S17. ^{1}H NMR spectrum of compound **5** (DMSO-d6, 500 MHz, 25^{0}C).

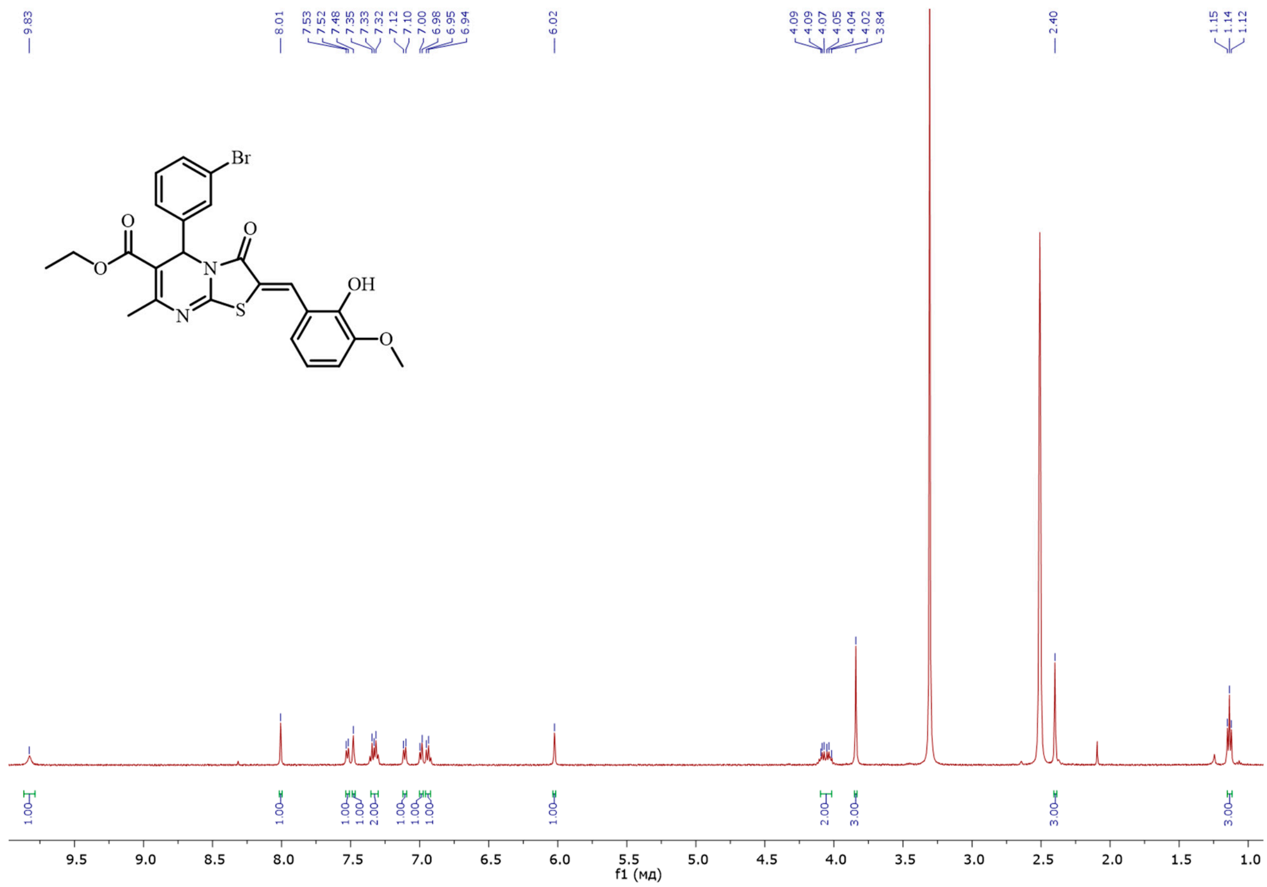

Figure S18. ^{13}C NMR spectrum of compound **5** (DMSO-d_6, 100 MHz, 25⁰C).

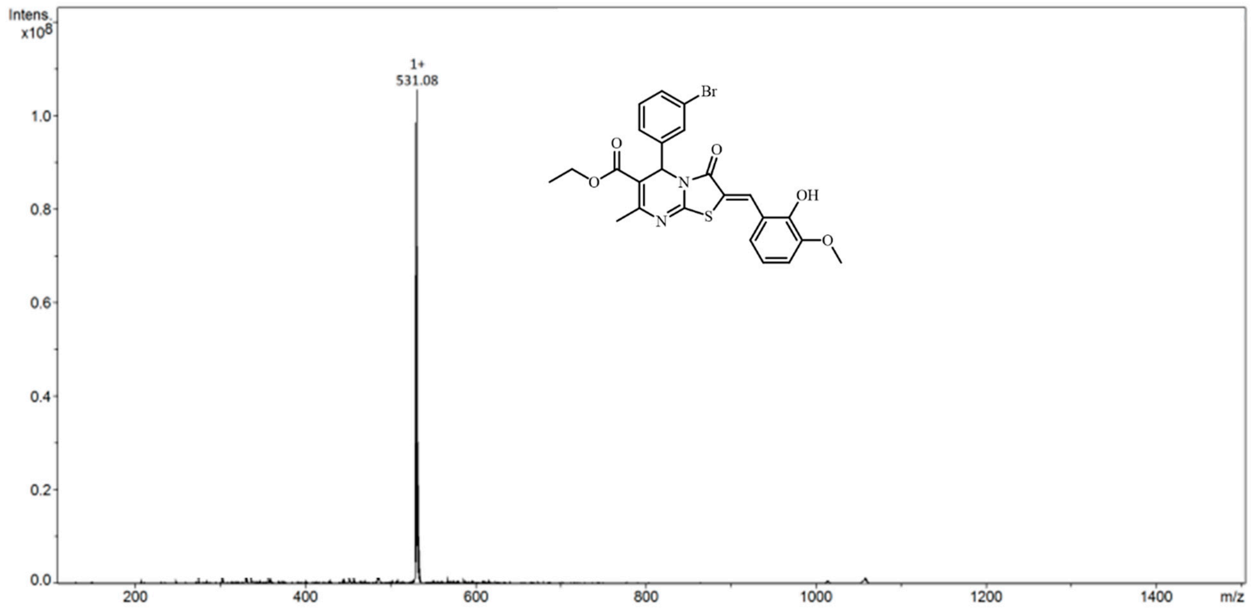

Figure S19. ESI MS spectrum of compound **5** (Ion Polarity: Positive).

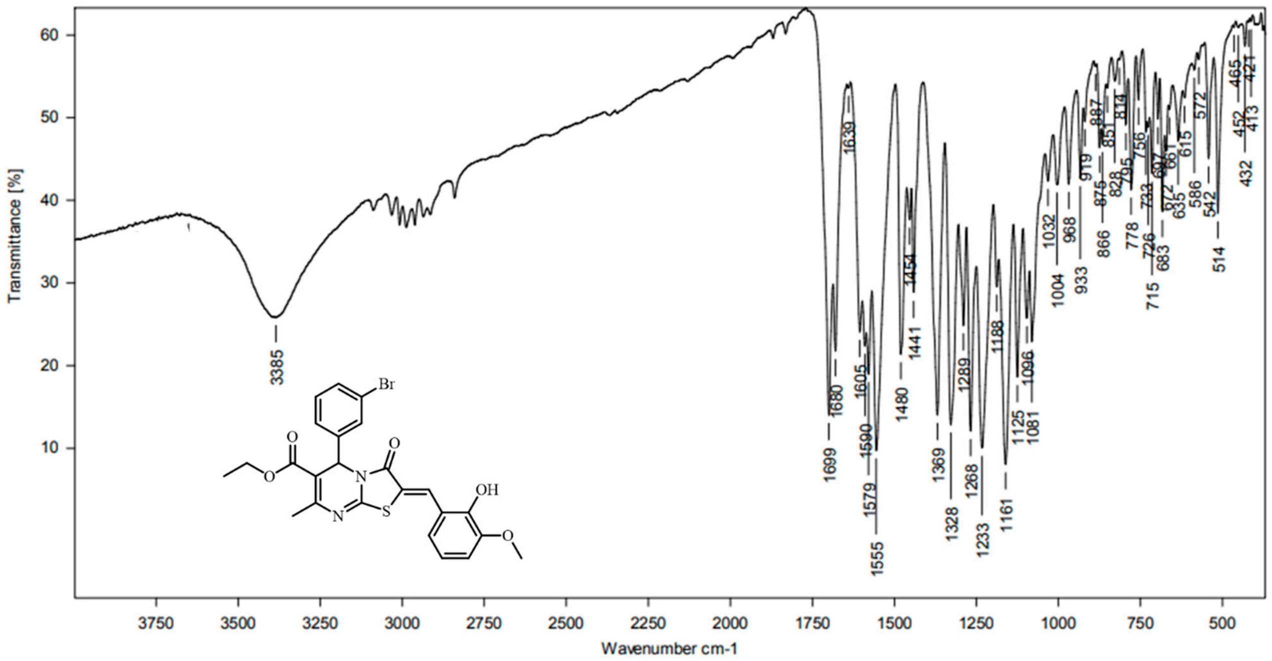

Figure S20. IR spectrum of compound **5** (KBr tablet).

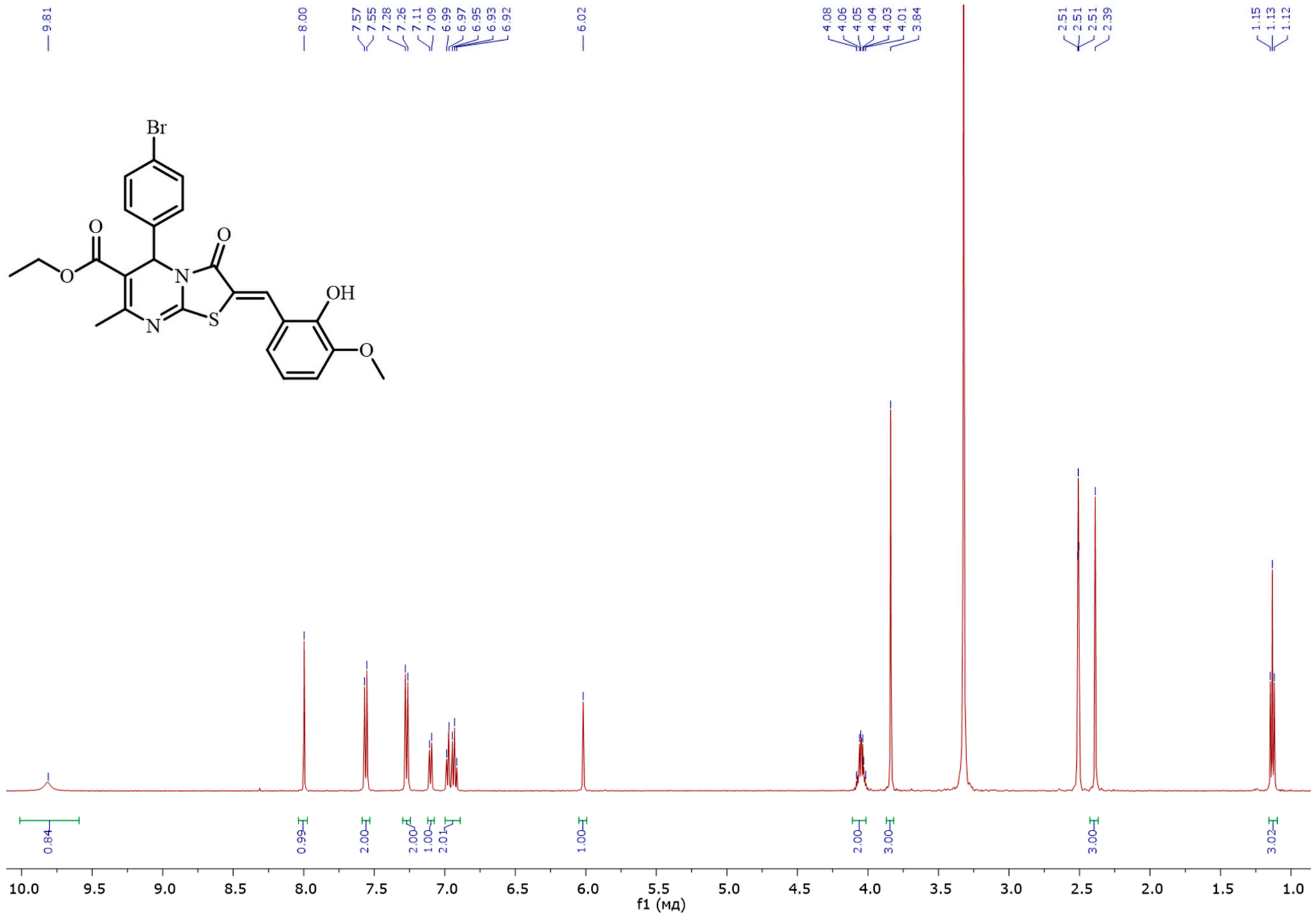

Figure S21. ^{1}H NMR spectrum of compound **6** (DMSO-d6, 500 MHz, 25^{0}C).

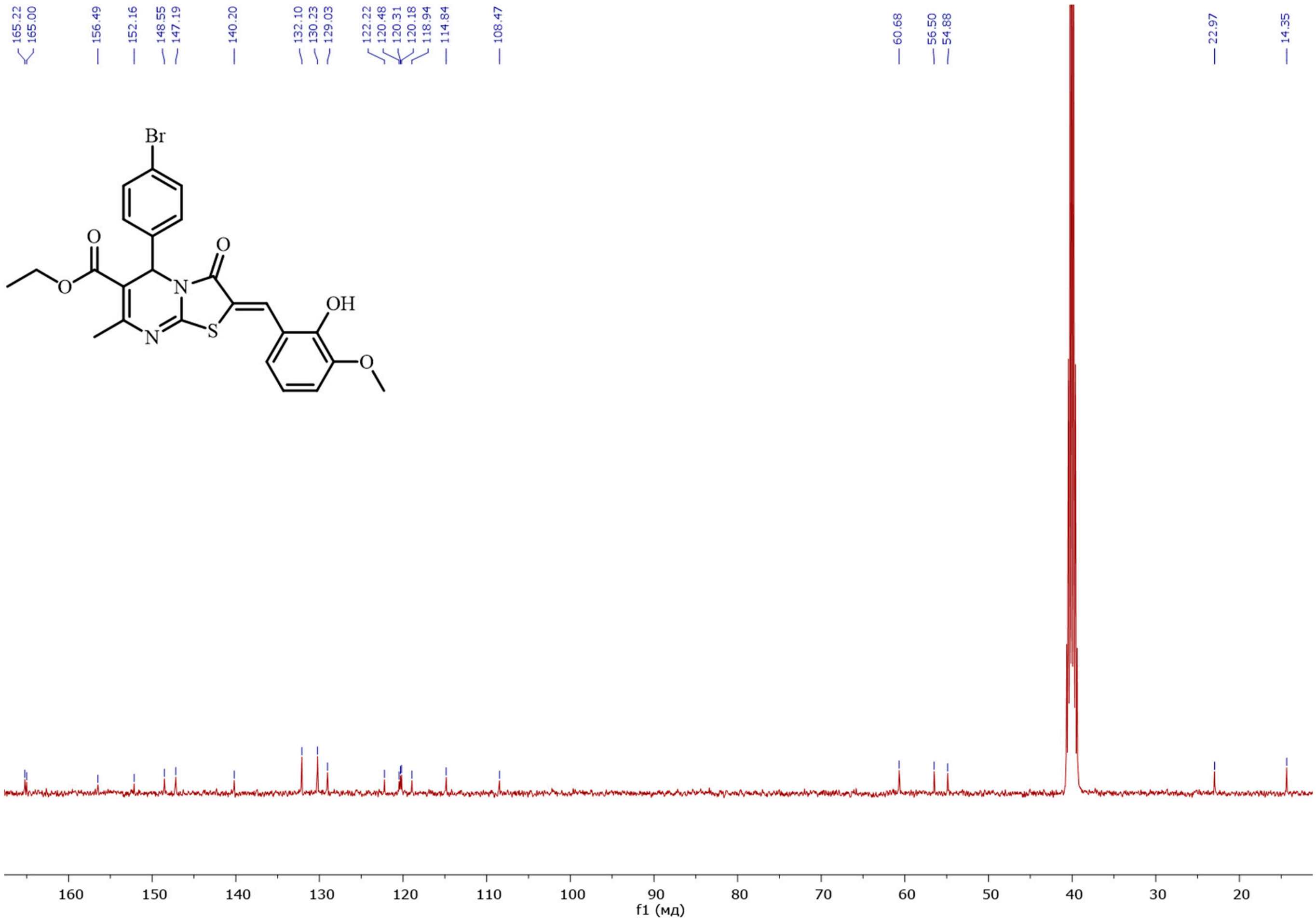

Figure S22. ^{13}C NMR spectrum of compound **6** (DMSO-d$_6$, 100 MHz, 25^{0}C).

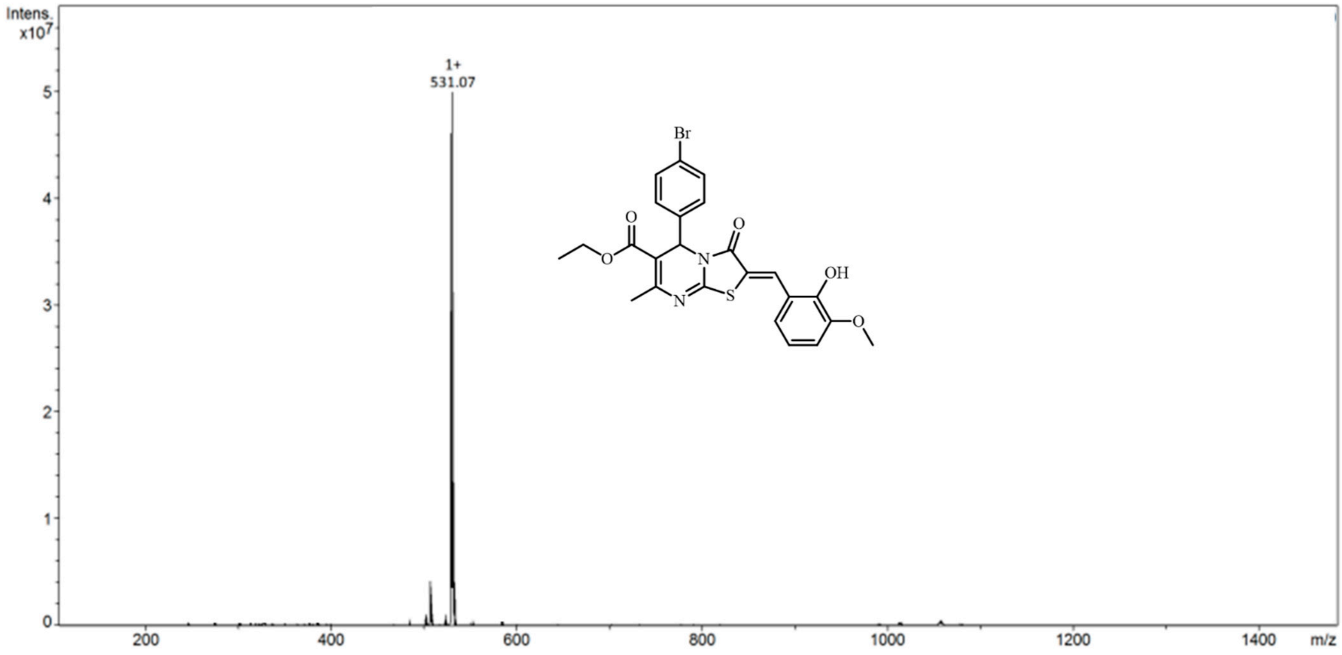

Figure S23. ESI MS spectrum of compound **6** (Ion Polarity: Negative).

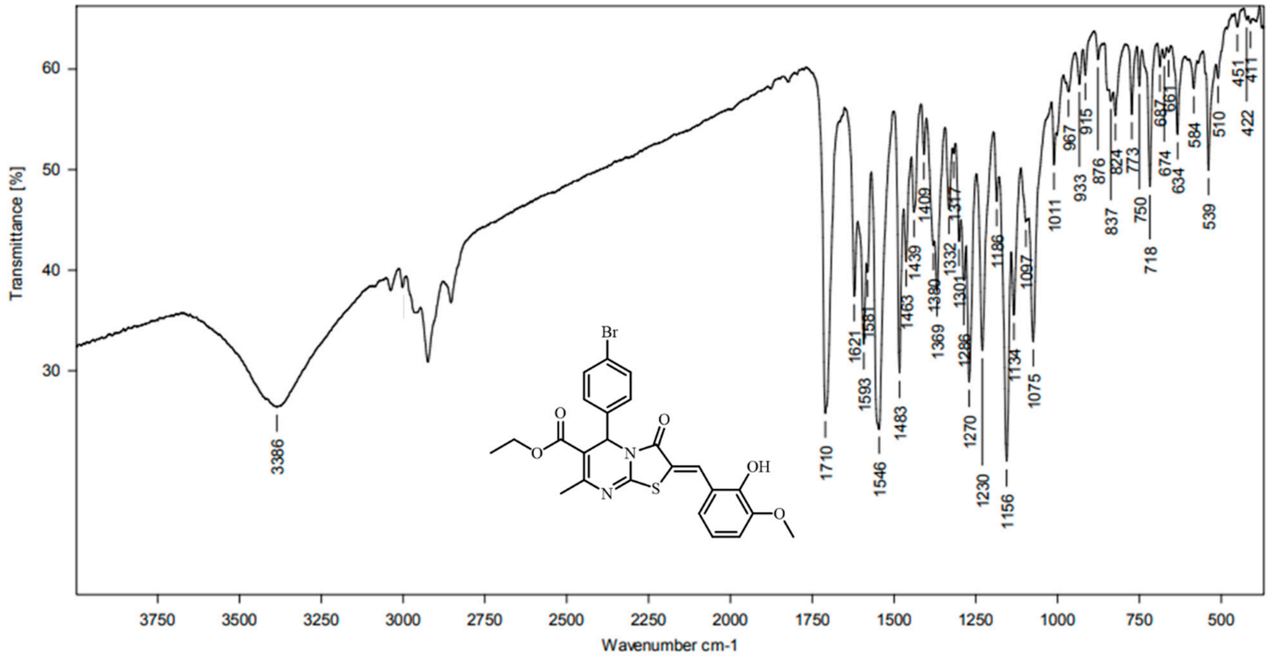

Figure S24. IR spectrum of compound **6** (KBr tablet).

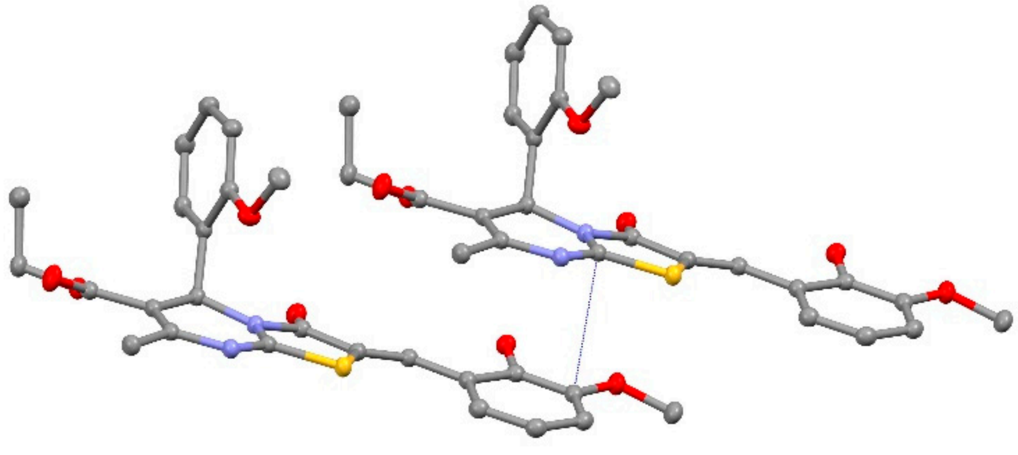

Figure S25. The π-staking involving the pyrimidine and 2-arylmethylidene groups for compound **3** ($d_{C9\text{-}C13}$ = 3.341 Å)

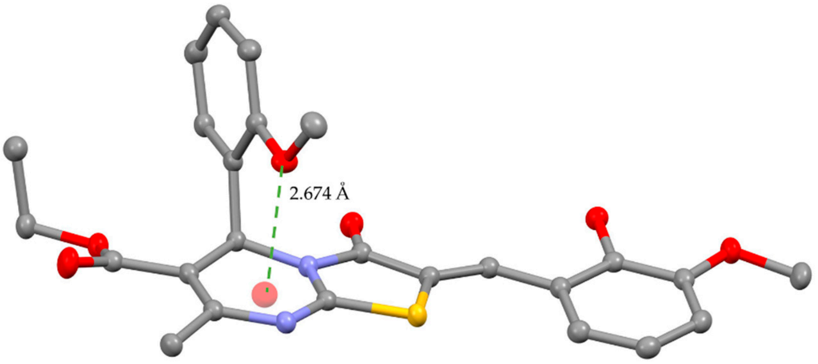

Figure S26. The n-π interaction of electron pair belonging to OMe group disposed in orto position of the phenyl substituent at C5-atom with the π-conjugated pyrimidyl moiety displaying the O19…C3N2 centroid distance equal to 2.674 Å.

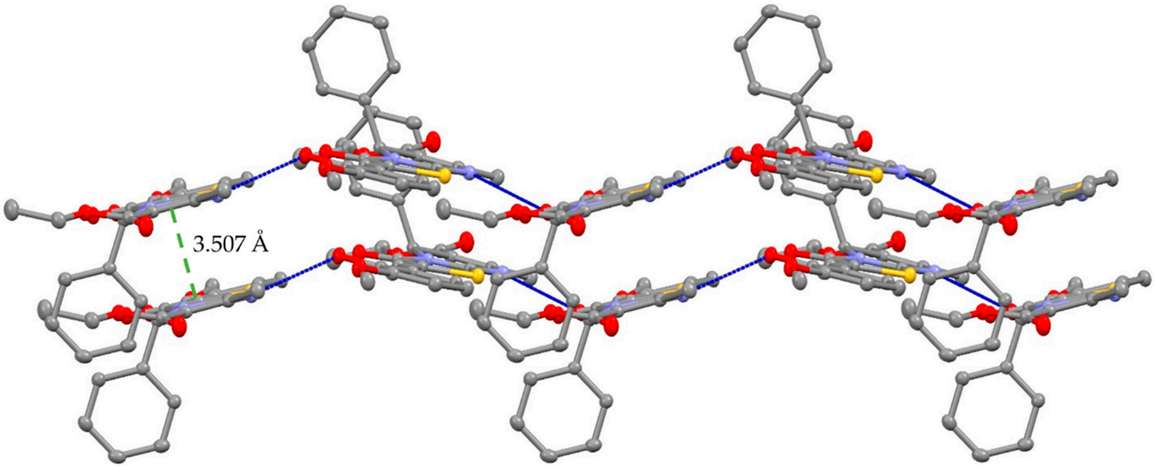

Figure S27. The 2D sheets formed by π-stacking of molecules for compound **4** in crystal.

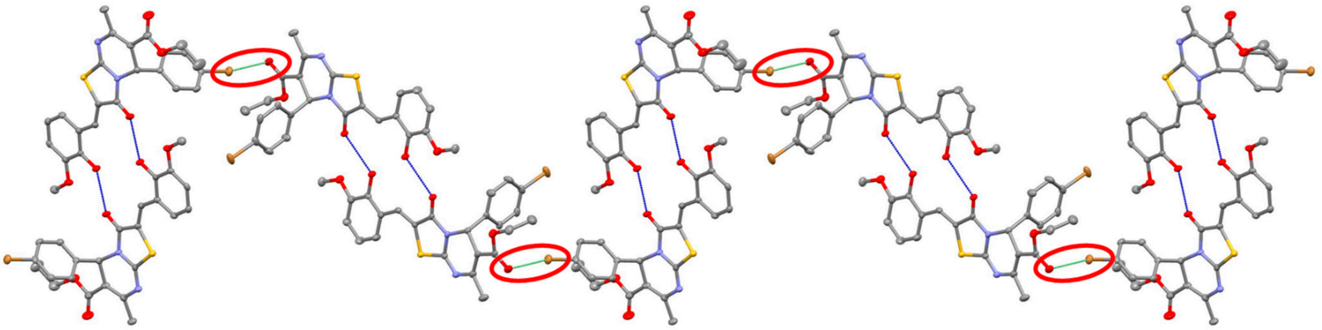

Figure S28. 1D halogen bonded chain of **6** is composed of two types of racemic dimers: the first one acts as bis-XB-donor due to two Br-atoms belonging two H-bonded molecules in the dimer structure and the another one behaving as bis-XB-acceptor offering two carbonyl ester O-atoms for interaction with Br-atoms.

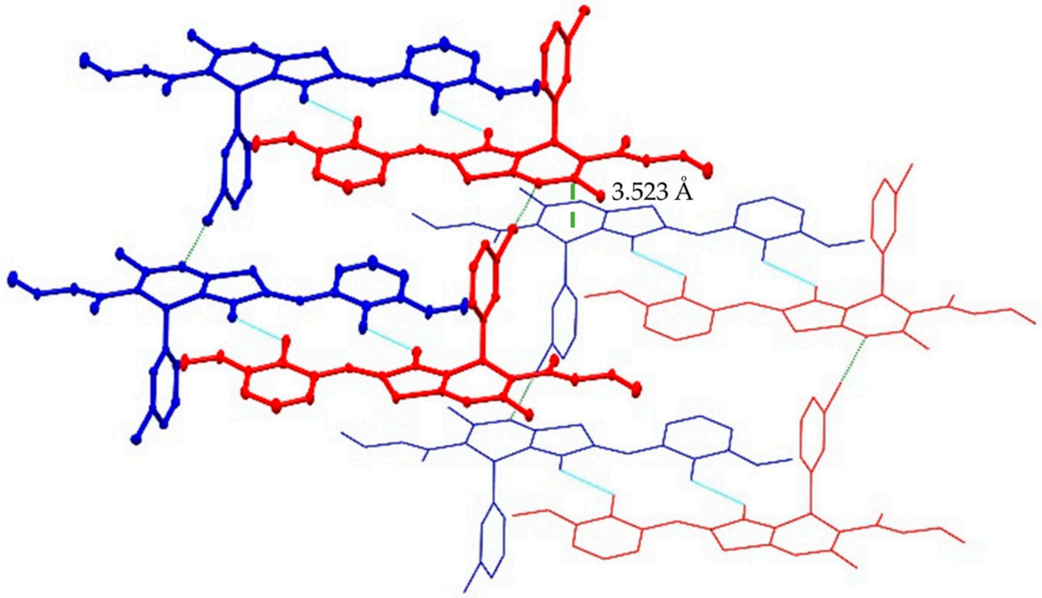

Figure S29. Part of the crystal packing demonstrating the arrangement of the layers resulting from the halogen bonding in crystal of **5**. R-, S-isomers, hydrogen and halogen bonds are colored in blue, red, light blue and green, respectively.

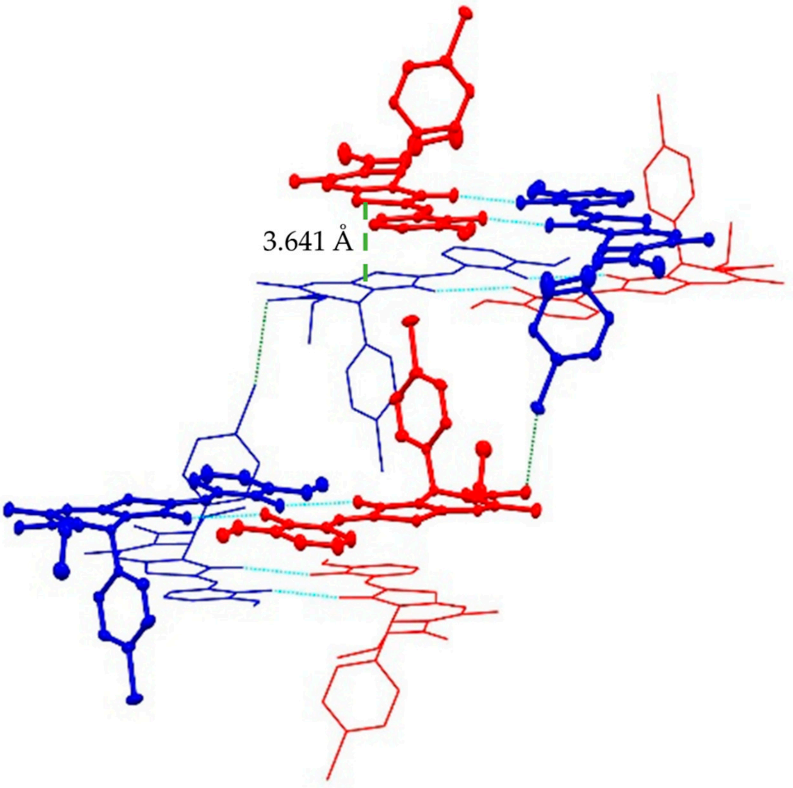

Figure S30. A part of the crystal packing showing the arrangement of layers due to halogen-bonding in crystal of compound **6**. R-, S-isomers, hydrogen and halogen bonds are colored in blue, red, light blue and green, respectively.

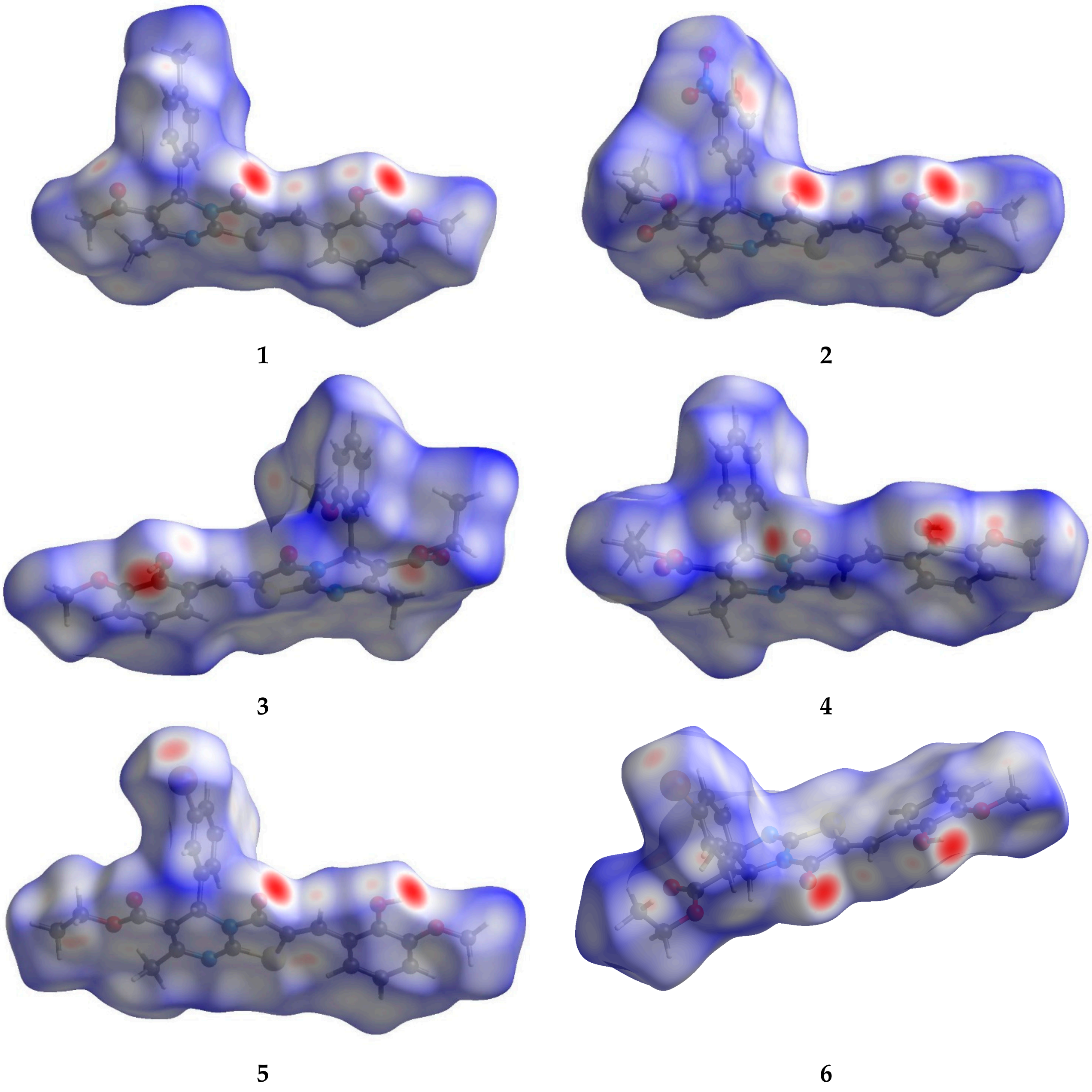

Figure S31. The standard 3D Hirshfeld surfaces of compound **1-6** mapped over d_{norm}.

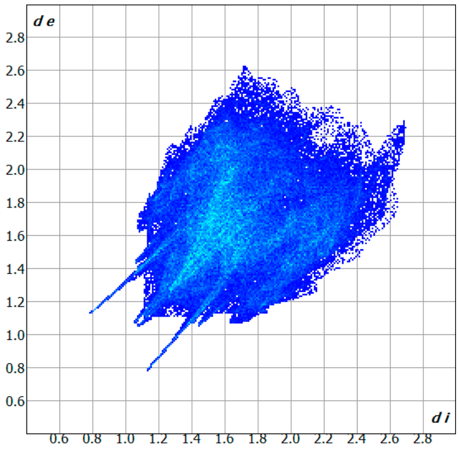

1

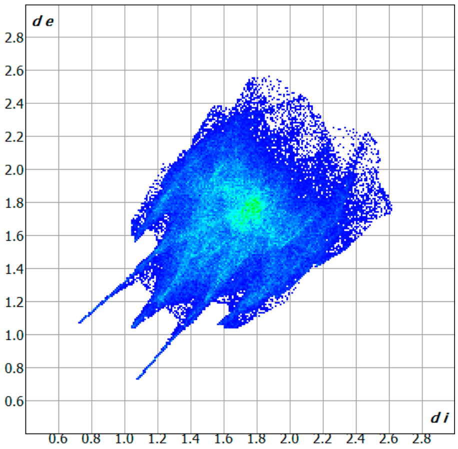

2

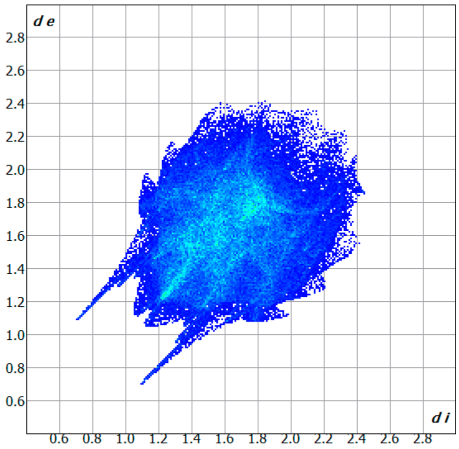

3

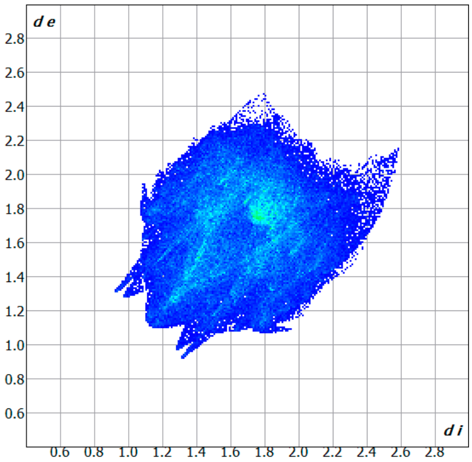

4

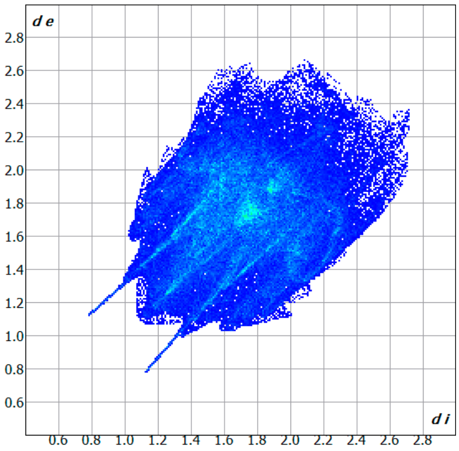

5

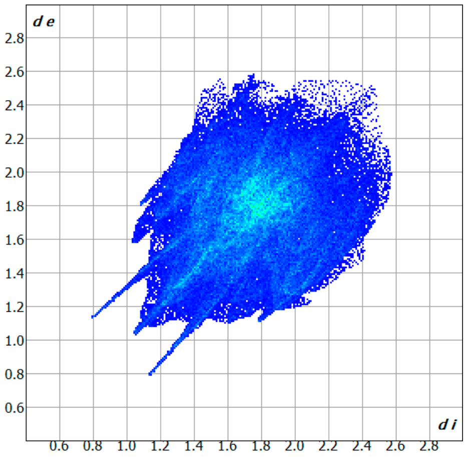

6

Figure S32. The overall two-dimensional fingerprint plots for compounds **1-6** showning the sum of the contacts contributing to the Hirshfeld surface.

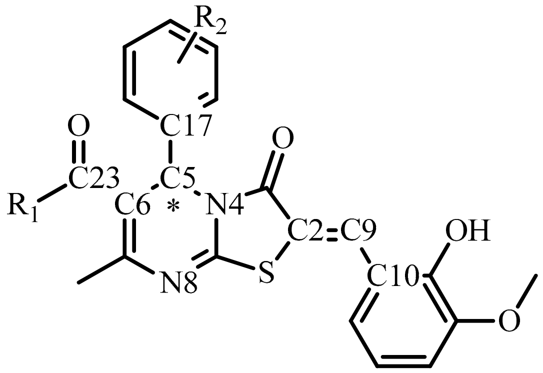

Table S1. Bond distances and angles of asymmetric C5 atom in studied compounds established by SCXRD.

Compound	1	2	2 DMSO	3	4	5	5 DMSO	6
d(C5-C6), Å	1.528(2)	1.524(2)	1.525(2)	1.526(2)	1.518(3)	1.522(2)	1.522(2)	1.525(4)
d(N4-C5), Å	1.471(2)	1.470(2)	1.470(2)	1.473(2)	1.475(3)	1.474(4)	1.474(4)	1.479(3)
d(N4-C3), Å	1.382(2)	1.382(2)	1.380(2)	1.392(1)	1.391(3)	1.391(2)	1.391(2)	1.389(3)
d(C5-C17), Å	1.520(2)	1.526(1)	1.527(2)	1.522(2)	1.524(2)	1.522(3)	1.522(3)	1.524(5)
∠C6-C5-N4, ⁰	108.5(1)	107.5(1)	108.68(8)	108.74(2)	108.2(1)	108.4(2)	109.34(3)	108.0(2)
∠C6-C5-C17, ⁰	112.7(1)	112.9(1)	110.05(8)	112.0(1)	111.8(1)	111.4(2)	114.68(2)	112.8(2)
∠N4-C5-C17, ⁰	110.2(1)	110.2(1)	110.91(8)	111.59(9)	110.2 (1)	119.6(2)	110.39(2)	109.5(2)

Table S2. Selected bond distances and dihedral angles for studied compounds established by SCXRD.

Compound	Dihedral angle between thiazolopyrimidine and the carbonyl group at C6 atom π-systems, ⁰	Dihedral angle between thiazolopyrimidine and phenyl substituent of benzylidene fragment π-systems, ⁰	d(C2-C9), Å	d(C9-C10), Å	d(C6-C23), Å
**1**	12.47	14.30	1.349(2)	1.452(2)	1.484(2)
**2**	12.36	9.45	1.351(2)	1.450(3)	1.476(3)
**2 DMSO**	11.86	9.47	1.347(2)	1.454(2)	1.472(2)
**3**	16.44	8.61	1.348(2)	1.453(2)	1.482(2)
**4**	18.97	2.77	1.347(3)	1.450(3)	1.484(3)
**5**	7.05	7.66	1.346(3)	1.448(4)	1.478(4)
**5 DMSO**	7.05	7.66	1.346(3)	1.448(4)	1.478(4)
**6**	8.81	9.47	1.353(4)	1.446(4)	1.480(4)

Table S3. H- bond distances presenting in crystals of studied compounds.

Compound	1	2	2 DMSO	3	4	5	5 DMSO	6
d(O3-O11), Å	2.810(1)	2.738(2)	2.733(1)	–	–	2.795(2)	2.795(2)	2.672(1)
d(O3-O12), Å	3.032(1)	2.899(2)	2.902(1)	–	–	3.071(1)	3.071(1)	3.162(2)
d(O11-N8), Å	–	–	–	2.763(1)	3.002(2)	–	–	–
d(O12-N8), Å	–	–	–	3.342(2)	3.849(2)	–	–	–
∠C11-O11-O3, ⁰	129.04(7)	126.9(1)	127.12(9)	–	–	129.60(2)	129.61(2)	132.4(2)
∠C12-O12-O3, ⁰	123.81(8)	123.8(1)	123.78(9)	–	–	122.36(1)	122.36(1)	117.70(1)
∠C11-O11-N8, ⁰	–	–	–	110.56(7)	131.4(1)	–	–	–
∠C12-O12-N8, ⁰	–	–	–	97.98(7)	106.6(1)	–	–	–

Table S4. Halogen bonds distances and angles presenting in crystals of compounds **5** and **6**.

**Compound**	**5**	**5 DMSO**	**6**
d(Br-N8), Å	3.036(2)	3.036(2)	-
d(Br-O24), Å	-	-	3.120(2)
∠C19-Br-N8, ^{0}	177.19(8)	177.19(8)	-
∠C21-Br-O24, ^{0}	-	-	166.0(1)

Table S5. The most important contributions (%) for the crystal packing in **1-6**.

**Type of interactions**	**1**	**2**	**3**	**4**	**5**	**6**
H···H	46.5	34.6	49.1	44.4	38.2	41.9
H···O/O···H	11.9	24.5	15.2	18.1	16.1	13.8
H···C/C···H	22.7	18.7	18.0	20.0	20.8	14.8
H···N/N···H	4.0	4.5	2.8	3.5	1.9	3.1
C···N/N···C	0.7	0.8	1.0	1.7	0.7	0.9
C···C	0.6	4.5	3.0	3.7	2.6	4.0
H···S/S···H	6.0	4.5	4.2	5.4	2.8	3.4
C···O/O ··C	5.0	3.3	3.1	1.9	1.3	1.3
O···O	1.1	2.1	0.6	0.2	0.7	1.4
N···O/O ··N	0.7	1.3	1.1	0.1	0.3	-
H···Br/Br ··H	-	-	-	-	7.6	9.4
N···Br/Br ··N	-	-	-	-	2.3	-
O···Br/Br ··O	-	-	-	-	-	0.7
C···Br/Br ··C	-	-	-	-	2.0	2.1
